# Habitat requirements affect genetic variation in three species of mayfly (Ephemeroptera, Baetidae) from South Africa

**DOI:** 10.3897/zookeys.936.38587

**Published:** 2020-05-28

**Authors:** Chantal L. Taylor, Nigel P. Barker, Helen M. Barber-James, Martin H. Villet, Lyndall L. Pereira-da-Conceicoa

**Affiliations:** 1 Department of Zoology and Entomology, Rhodes University, Somerset Street, Makhanda (Grahamstown), 6140, South Africa; 2 Department of Plant and Soil Sciences, University of Pretoria, Pretoria, 0028, South Africa; 3 Department of Freshwater Invertebrates, Albany Museum, Somerset Street, Makhanda (Grahamstown), 6140, South Africa; 4 Department of Natural Sciences, National Museums Scotland, Edinburgh, Chambers Street, EH1 1JF, United Kingdom

**Keywords:** cytochrome oxidase 1, genetic diversity, habitat specialisation, haplotype, phylogeography, mayfly

## Abstract

This study investigates genetic diversity in three species of Ephemeroptera, one eurytopic and therefore widespread (*Afroptilum sudafricanum*) and two stenotopic and thus endemic (*Demoreptus natalensis* and *Demoreptus capensis*) species, all of which co-occur in the southern Great Escarpment, South Africa. Mitochondrial DNA was analysed to compare the genetic diversity between the habitat generalist and the two habitat specialists. *Afroptilum sudafricanum* showed no indication of population genetic structure due to geographic location, while both *Demoreptus* species revealed clear genetic differentiation between geographic localities and catchments, evident from phylogenetic analyses and high F_ST_ values from AMOVA. In addition, the phylogenetic analyses indicate some deeper haplotype divergences within *A. sudafricanum* and *Demoreptus* that merit taxonomic attention. These results give important insight into evolutionary processes occurring through habitat specialisation and population isolation. Further research and sampling across a wider geographic setting that includes both major mountain blocks of the Escarpment and lowland non-Escarpment sites will allow for refined understanding of biodiversity and associated habitat preferences, and illuminate comparative inferences into gene flow and cryptic speciation.

## Introduction

Greater genetic diversity within a lineage is regarded as increasing its resilience to environmental change (Jump et al. 2009, Razgour et al. 2019), which gives contemporary relevance to insights into the mechanisms shaping genetic diversity of populations. Genetic diversity between populations is, in part, a reflection of their members’ dispersal activity through space and time (Slatkin 1985, Bohonak 1999, Avise 2009). Theoretically, if widespread intermigration between populations of a species occurs, then levels of genetic differentiation will be relatively low, whereas if dispersal is restricted by physical barriers or limitations to mobility, then genetic differentiation is likely to be higher (Slatkin 1993). The relationship between dispersal ability and genetic population structure of a species can provide important insights into micro-evolutionary processes, phylogeography (Hanski and Gaggiotti 2004, Avise 2009), and resilience to environmental change.

Aquatic insects have a winged adult stage that is generally considered to have relatively strong dispersal ability (Hughes and Mather 1995, Bunn and Hughes 1997). This is reflected in the ability of stream organisms to recover from disturbance (Wallace 1990, Yount and Niemi 1990) and the widespread geographic distribution of many aquatic species across catchments. Consequently, many such insects show low levels of genetic differentiation among populations, both within and between catchments, attributed to the extensive dispersal of adults by flying (Schmidt et al. 1995, Hughes et al. 1998, 2000, Miller et al. 2002, Monaghan et al. 2002, Pereira-da-Conceicoa et al. 2012, Gattolliat et al. 2018). Despite the apparent mobility of these species, their need for persistent waters for breeding tends to fragment their distribution into metapopulations (Avise 2009). The patchiness of lakes, the linear, unidirectional, hierarchical character of rivers, and the topographical structure of catchments tend to structure the dispersal of aquatic organisms between breeding sites or local populations (Wishart et al. 2003, Kaltenbach and Gattolliat 2018). The population genetic variance of certain species is structured significantly according to drainage basin, especially in mountainous landscapes with rugged topography (Hughes et al. 1999, 2003, Wishart and Hughes 2001, 2003, Monaghan et al. 2002, Price et al. 2010, Toussaint et al. 2013, 2014, Barber-James and Pereira-da-Conceicoa 2016). Ecologically, aquatic habitats within terrestrial landscapes can therefore be conceptualised as functional islands for some aquatic organisms.

Genetic variation between populations is related to the ability of their members to disperse, and a high degree of genetic structure has been observed among populations of some South African winged aquatic (Wishart and Hughes 2001, 2003) and terrestrial (Price et al. 2007, 2010) insects. This has been attributed to habitat-specificity that imparts a high cost to unsuccessful dispersal, so that stronger associations with restricted habitats, such as particular aquatic conditions, result in increasingly limited potential for successful dispersal (Price et al. 2007). Aquatic invertebrate species, including Ephemeroptera, show varied degrees of habitat-specificity, with some species being completely restricted to a certain habitat and others occurring in a range of habitat types (Barber-James and Lugo-Ortiz 2003).

The aim of this study is to use three model species of mayfly to test the hypothesis that habitat-restricted taxa have greater phylogeographical structure than habitat-generalist species. *Afroptilum sudafricanum* Lestage is a common, widespread African species, occurring in a range of ecological conditions, including different flow regimes and a wide altitude range (Barber-James and Lugo-Ortiz 2003). *Demoreptus natalensis* Crass and *Demoreptus capensis* Barnard have very specific habitat requirements, being most commonly found on rock faces associated with waterfalls in fast-flowing mountain streams (Barber-James and Lugo-Ortiz 2003).

## Materials and methods

### Study region

The southern Great Escarpment forms an 800-km-long stretch of mountain complexes extending from the Nuweveldberge in the west to the Eastern Cape Drakensberg in the east. Ancient erosional features divide the mountains into five main blocks that range in altitude from 1 600–3 000 m a.s.l., making the area interesting for study of dispersal-limited groups.

### Taxon sampling

Nymphs of *A. sudafricanum*, *D. capensis*, and *D. natalensis* were collected from 21 rivers in the Eastern Cape Great Escarpment, relating to 12 study areas within the Escarpment and non-Escarpment sites (Table 1). An additional six rivers were sampled for *A. sudafricanum* in lower-altitude (non-Escarpment) areas in the Eastern Cape and KwaZulu-Natal (Table 1). All specimens were preserved in 80% ethanol.

**Table 1.** Collecting localities (Site and river name) and non-zero sample sizes for each species from each site. The GenBank sequence accession numbers for each sample are listed in Suppl. material 1.

Locality	Longitude/Latitude	*A. sudafricanum*	*D. capensis*	*D. natalensis*	*Demoreptus* sp.
**Escarpment sites**					
**Eastern Cape Drakensberg**					
Barkley East 1: Diepspruit	-30.751, 27.546	3	1		
Barkley East 2: Diepspruit	-30.757, 27.552	3			
Barkley East 3: Diepspruit	-30.718, 27.54	3			1
Barkley Pass 1: Marais Hoek	-31.215, 27.686	3			
Barkley Pass 4: Ben Wyvie	-31.173, 27.971	3		3	
Barkley Pass 5: Lymore Lodge	-31.172, 27.854		2		
Rhodes 1: Hawkshead	-30.676, 27.884	3	2		
Rhodes 2: Tiffindell	-30.674, 27.904	3	1		
Rhodes 3: Tenahead	-30.696, 28.150	3		1	
Maclear 1: Vuvu River	-30.603, 28.216	5			
**Stomberg**					
Stomberg 3: Lana River	-31.163, 26.602	3			
Stomberg 4: Lemonfountain	-31.416, 26.842	3			
**Winterberg-Amatole**					
Elansberg 1: Elandsberg	-32.506, 26.903	3			
Winterberg 1: Fanella falls	-32.363, 26.385	2		3	
Winterberg 2: Fanella falls	-32.380, 22.967	3			
Winterberg 3:	–	5			
**Sneeuberg**					
Sneeuberg 1: Fish River	-32.227, 24.954	2			
Sneeuberg 2: Melkriver	-32.243, 24.941	2	3	3	
Kamdeboorberg 1: Buffelsrivier	-32.177, 24.016	3		2	
Kamdeboorberg 3: Waterkloof	-32.353, 23.890	2	2		
**Nuweveldberge**					
Nuweveldberge 1: Maijiesvlei	-32.102, 22.636	1			
**Non-Escarpment sites**					
**Grahamstown**					
Grahamstown CR: Coleridge River	-33.349, 26.618	2			
Grahamstown KP: Kap River	-33.351, 26.858	5			
Grahamstown KR: Kowie River	-33.349, 26.560	5			
Grahamstown PM: Palmiet River	-33.370, 26.476	5			
**KwaZulu-Natal**					
KwaZulu-Natal KK: Karkloof River	-29.338, 30.307	5			
KwaZulu-Natal LR: Lions River	-29.492, 30.108	5			
KwaZulu-Natal UM: Umgeni River	-29.477, 30.261	1			
		86	11	12	1

A related species of Baetidae, *Baetis rhodani* Pictet, was used as the outgroup for phylogenetic analyses, and relevant sequence data (Rutschmann et al. 2014) were obtained through Genbank (Benson et al. 2012) for both cytochrome c oxidase subunit I (COI) (KP438135 and KP438160) and 16S rRNA (16S) (KP438109 and KP438119) gene regions.

### DNA extraction, amplification, and sequencing

DNA was extracted using the Invisorb Spin Tissue Mini Kit following manufacturer’s protocol (Invitek, Berlin, Germany). Extraction was non-destructive, using internal body digestion, which ensured the preservation of the exoskeleton for future morphological analysis (housed in the Albany Museum, Makhanda, South Africa, along with additional material that is stored in the collection, listed under the GEN catalogue.)

Two mitochondrial gene regions were amplified: cytochrome c oxidase subunit I (COI) and small subunit ribosomal RNA (16S). A 528-bp section of the COI regions of *D. natalensis* and *D. capensis* was successfully amplified with the standard ‘universal’ primer pair, LCO1490 and HCO2198 (Folmer et al. 1994), which worked with only limited initial success with *A. sudafricanum*. A new forward primer (5’–GGT GGA TGG GCA GGA ATG GTA GGA–3’) was designed and used with HCO2198 to successfully sequence the remaining samples of *A. sudafricanum*. The 16S region was amplified with the primer pair 16Sar (5’–CGC CTG TTT ATC AAA AAC AT–3’) and 16Sbr (5’–CCG GTC TGA ACT CAG ATC ACG T–3’) (Palumbi 1996). However, these primers proved problematic for the *Demoreptus* samples, and this region is thus excluded from subsequent analyses for this taxon.

The polymerase chain reaction (PCR) was performed in a 50 μl volume using the following thermal regime: 95 °C for 5 min, 35 cycles of 95 °C for 45 s, 50 °C for 45 s, and 72 °C for 90 s, followed by a final extension period of 72 °C for 5 min. PCR amplifications were checked for the presence of amplified PCR products by gel electrophoresis (0.5% agarose gel stained with SYBR green) and viewed with a UV-transilluminator. Successful PCR products were cleaned up using the Invisorb PCRapace® Quick purification kit (Invitek, Berlin, Germany) and cycle-sequenced in both directions using the primers used for amplification, the ABI Big Dye Sequencing kit v.3.1 (following manufacturer’s instructions (Applied Biosystems)), and a ABI Genetic Analyzer 3500 (Applied Biosystems).

Sequence trace files were assembled and edited using Sequencher 3.0 (DNA sequence analysis software, Gene Code Corporations, Ann Arbor, MI USA, http://www.genecodes.com). The sequences were then aligned in MEGA v.6 (Tamura et al. 2013) using the ClustalW algorithm and subsequently each non-synonymous mutation was manually cross-checked in the original trace files.

### Phylogenetic analyses

Each gene was tested for substitution saturation using plots of transitions and transversions against F84 distance in DAMBE v7.0.58 (Xia et al. 2003, Xia and Lemey 2009, Xia 2017).

Congruence between the COI and 16S datasets was assessed using the partition homogeneity test (PHT) in PAUP* (Swofford 2002) with 1000 replicates to verify that the gene sections could be combined for analysis.

Bayesian Inference (BI) analyses were conducted with MrBayes v.3.1.2 (Huelsenbeck and Ronquist 2001) using the GTR+I+G model since it is the most complex model, allowing the nesting of simpler models that could be estimated through the Bayesian sampling. Each analysis comprised two independent runs with random starting trees and four chains (three heated and one cold) each, sampled every 200 generations for 20 million generations per run. The cumulative sample sizes were plotted against the likelihood scores and tree length using Tracer v1.7.0 (Rambaut et al. 2018), to ascertain when the analysis reached stationarity after the first 10% of the trees were discarded as burn-in. The analysis was run on the CIPRES Science Gateway (Miller et al. 2010) using default parameters for variables not mentioned above.

Maximum likelihood (ML) analyses were conducted with 2 000 bootstrap replicates using the GARLI (Genetic Algorithm for Rapid Likelihood Inference) on XSEDE via the CIPRES (Cyberinfrustructure for Phylogenetic Research) Science Gateway v3.3 (Miller et al. 2010), which is supported by the San Diego Supercomputer Center (SDSC) and the University of California (UC San Diego). Models of molecular evolution for each dataset were selected using the Akaike information criteria (AIC) as implemented by jModeltest 2.1.6 (Darriba et al. 2012) (Table 2). The COI and COI+16S ML phylograms were compared and presented using Phylo.io software (Robinson et al. 2016).

Parsimony analyses were performed in PAUP* version 4.0b10 (Swofford 2002) using the heuristic search option with 100 random addition replicates. A search with TBR (Tree Bisection and Reconnection) branch-swapping was used to find the approximate length of the shortest trees, with one tree kept with each random addition. To investigate nodal support, all of the trees from this search were used as starting trees for a second heuristic search with MAXTREES set to 5 000. The *Demoreptus* analysis used FASTBOOTSTRAP with 10 000 replicates.

**Table 2.** Data characteristics and summary of the parsimony analysis. The number of specimens with sequence data (ntax), total number of base pairs (bp), parsimony informative (# Pi), and percent parsimony informative (% Pi) is reported. The results of the parsimony are summarised with the number of trees retained (# trees), tree length (score), Consistence Index (CI) and Retention Index (RI). The summary of the models for the Maximum Likelihood analysis (ML) selected by jModeltest.

Species	Dataset	ntax	Characters				Parsimony analysis				Model	
			bp	#Var	# Pi	% Pi	# trees	Score	CI	RI	ML analysis	BI analysis
*A. sudafricanum*	COI	88	649	217	192	29.6	5 000	421	0.601	0.932	GTR+I+G	GTR+I+G
	COI+16S	88	1191	380	336	28.2	5 000	645	0.662	0.939	TIM3+I+G	GTR+I+G
*Demoreptus* spp.	COI	24	528	164	159	30.1	8	302	0.745	0.922	TVM+G	GTR+I+G

### Phylogeographical structure and variation

Molecular diversity was investigated using the COI datasets. The number of variable sites (S), number of haplotypes (Hap) and haplotype diversity (Hd), Nucleotide diversity (p) and neutrality tests (Tajima’s D and Fu’s F_S_) were calculated in DNAsp (Rozas et al. 2017). Population structures within each species were estimated using one-level Analyses of Molecular Variance (AMOVA) in ARLEQUIN ver. 3.5.2 (Excoffier and Lischer 2010). F_ST_ (fixation index) values were calculated between localities to determine whether putatively conspecific populations differed significantly in their genetic composition. For all AMOVA analyses (listed in Table 4), *a priori* groups were defined by each site where the insects were collected. Haplotype networks were illustrated with a median-joining network (MJN) algorithm (ε = 0) (Bandelt et al. 1999) using the software PopART v. 1.7 (Leigh and Bryant 2015) to analyse haplotype genealogy.

## Results

### Data characteristics

COI sequences (649 bp) were obtained from 86 individuals and 16S sequences (542 bp) obtained from 59 individuals of *A. sudafricanum*. Shorter (528 bp) COI sequences were obtained for 24 *Demoreptus* individuals (for *D. natalensis*, *N* = 12; *D. capensis*, *N* = 11; unidentified, *N* = 1). DNA characteristics for each gene dataset are summarised in Table 2. The Partition Homogeneity Test for incongruency (Swofford 2002) showed that the combined COI and 16S datasets were not significantly incongruent (P = 0.3890) and could therefore be combined for analysis. The COI+16S molecular dataset consisted of 59 specimens and 1191 nucleotides including the outgroup. No evidence of saturated substitution was found for either gene (data not shown).

### Phylogenetic analyses

**Figure 1.**
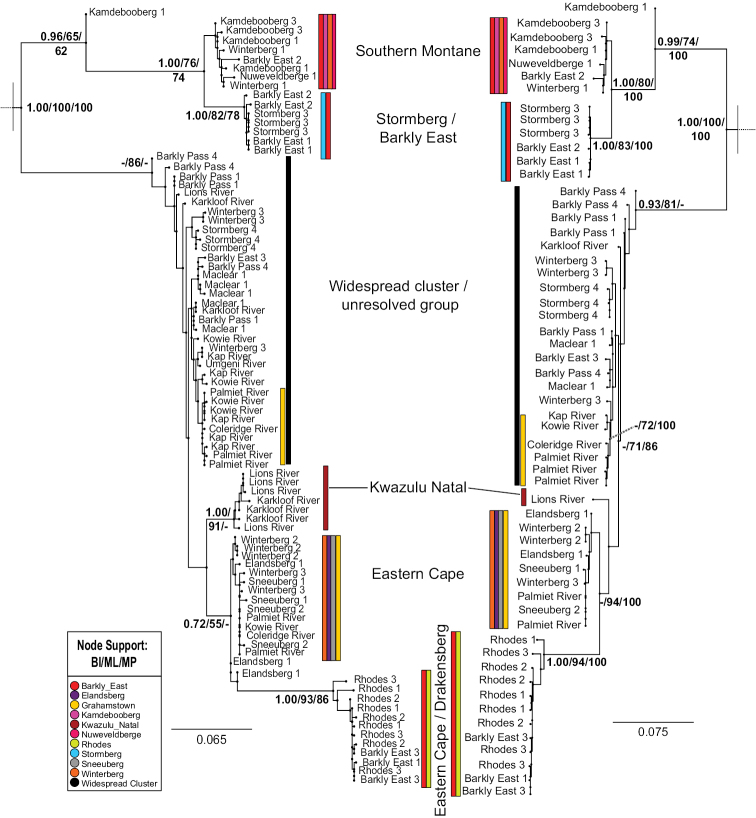
Bayesian inference phylograms of *A. sudafricanum* for gene markers COI (left) and COI + 16S (right). Support for major nodes shown in the order Bayesian Inference / Maximum Parsimony / Maximum Likelihood (BI/ML/MP). Bars next to clades refer to distinct clades that are colour-coded according to the study areas found within that clade (see colour legend), except for the widespread grade which is designated by a solid black line. Branches bearing outgroups have been omitted to save space and their position is depicted by a dashed line.

The parsimony analyses’ results are summarised in Table 2. Phylogenetic analyses of the habitat generalist *A. sudafricanum* consistently retrieved six distinct clades and an unresolved grade of specimens (referred to hereafter as the “widespread grade”) from a wide range of sites for all analyses (Fig. 1). The tree comparison shows that the relationships between these clades in the analysis of the CO1 data and the CO1+16S data sets were consistent, with improved support for the combined dataset. The well-supported clades did not conform to the mountain blocks described in Table 1 and included specimens from across these ranges. The clades were roughly separated into overlapping geographic groups: Southern Montane, Stormberg/Barkly East, and Eastern Cape, while more restricted geographic areas included KwaZulu-Natal and Eastern Cape Drakensberg, the latter clade showing a longer stem branch compared to other clades (Fig. 1).

**Figure 2.**
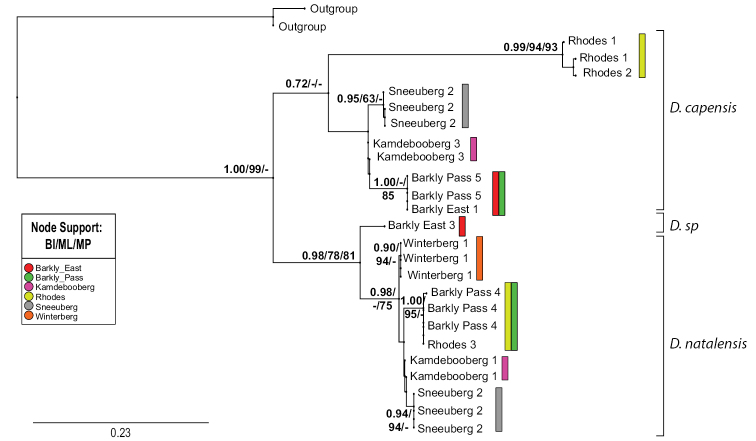
Bayesian inference phylogram of *Demoreptus* spp for the COI gene marker. Support for major nodes is shown in the order Bayesian Inference / Maximum Parsimony / Maximum Likelihood (BI/ML/MP). Bars next to clades refer to distinct clades that are colour-coded according to the study areas found within that clade (see colour legend). *Baetis rhodani* Pictet was used as the outgroup.

The phylogenetic analysis of the habitat-restricted *D. capensis* and *D. natalensis* clearly indicated strong genetic structure corresponding to geographic location (Fig. 2). Both species had genetically distinct populations with strong support from parsimony, Bayesian and maximum likelihood analyses. The clades found for *D. capensis* and *D. natalensis* were more closely aligned with the mountain ranges described in Table 1 and appear to be site-restricted, apart from one instance where individuals of *D. natalensis* from Rhodes and Barkly Pass fell into the same well-supported clade. For *D. capensis* the Rhodes clade had a long branch and is clearly distinct from the other clades, which is noteworthy considering the close geographic proximity to Barkly East, which formed a separate well-supported clade nested with other clades (Fig. 2). This pattern was not apparent in *D. natalensis*, where the Barkly East specimen was clearly separate from the *D. natalensis* clade; morphological re-examination suggests that it does not belong to any described *Demoreptus* species.

### Population genetics

**Figure 3.**
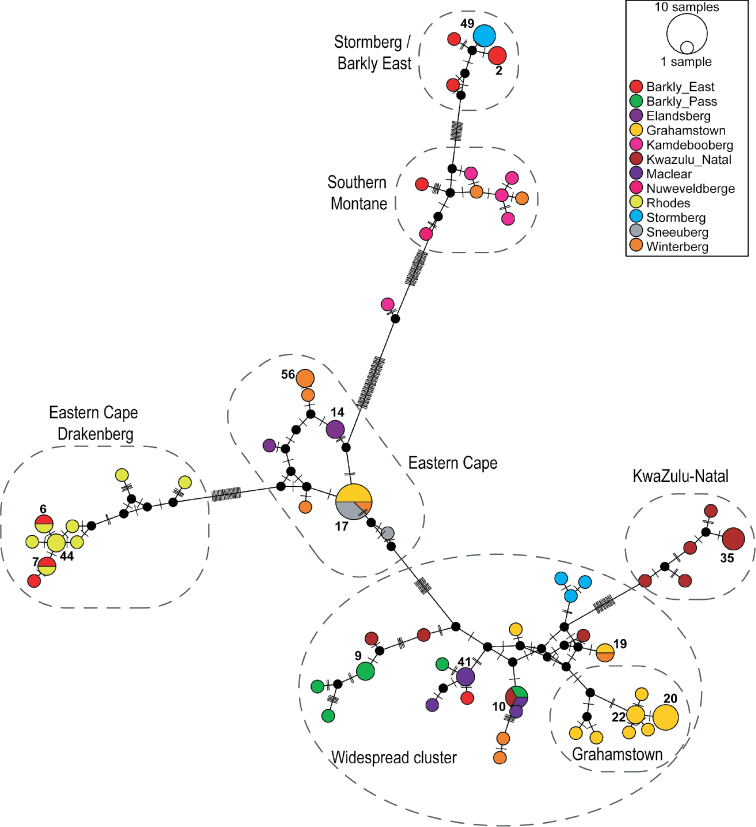
Median-joining network of *A. sudafricanum* based on COI haplotypes generated in this study. The network was estimated using the median-joining algorithm in PoPArt v.1.7 with epsilon = 0. Each circle represents a different haplotype and the size of a circle correlates with the number of individuals assigned to that haplotype. Only haplotypes found in more than one sample are numbered. Colours indicate the geographic origin of sequences; black dots indicate unsampled or extinct haplotypes.

**Figure 4.**
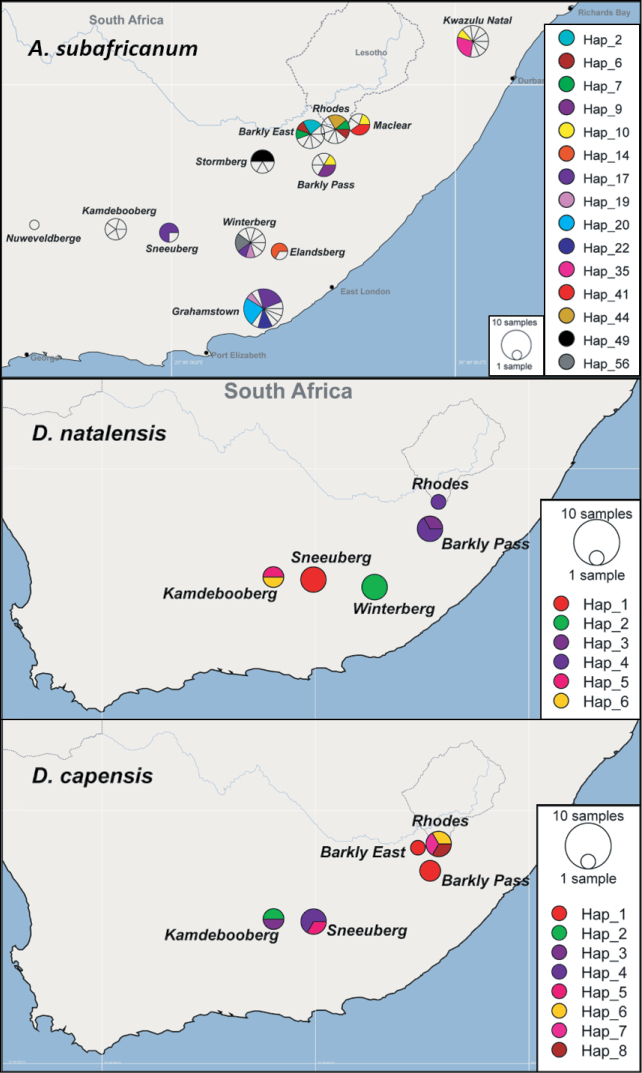
Distribution of *A. sudafricanum*, *D. natalensis* and *D. capensis*COI haplotypes across the study area. The map shows the study areas defined in Table 1, and the pie charts indicate the haplotype composition of the population from each area. Each colour represents a shared haplotype found across the study area; private haplotypes (singletons found in the samples from one particular population and are absent in the samples from other populations) are represented as clear sections within the pie charts.

MJN analysis collapsed the 86 *A. sudafricanum*COI sequences into 60 haplotypes (Fig. 3, Table 3), 45 of which were singletons or private haplotypes. Haplotype 17 was the most abundant (N = 8) and occurred in three of the 12 study areas (Fig. 4), which included non-Escarpment Grahamstown and two main mountain Escarpment blocks (Sneeuberg and Winterberg–Amathole). Haplotype 20 was next-most-abundant (N = 4) and exclusive to non-Escarpment Makhanda (= Grahamstown). Haplotype 10 (N = 3) was found in one non-Escarpment site (KwaZulu-Natal) and one main mountain Escarpment block (Eastern Cape Drakensberg, in two study areas: Barkly Pass and Maclear; Figs 3, 4). Haplotypes were clustered according to a broad geographical structure, which correspond to clades from the phylogenetic analyses (Fig. 1). The numerous missing mutational steps in the haplotype network (Fig. 3) suggest that more sampling is needed for some clusters, particularly between sites that are separated by long sampling gaps (for example, the non-Escarpment sites). Other clusters that are separated by numerous missing intermediates could represent cryptic species or relict lineages that have re-joined the metapopulation (Hinojosa et al. 2019) (encircled with dashes in Fig. 3). The divergent Haplotype 27 from the Kamdebooberg did not cluster with the other haplotypes from the same area and may represent such a lineage. The widespread grade showed little geographic structure, and all haplotypes from Stormberg (Hap 51, 52 and 53) grouped together exclusively, otherwise all other sites are mixed.

**Table 3.** Haplotype characteristics and Neutrality tests for *A. sudafricanum*, *D. capensis* and *D. natalensis*.

Species	Haplotype characteristic		
	Number of haplotypes (Hap)	Nucleotide diversity (Pi)	Number of variable sites (S)
*A. sudafricanum*	60	0.07508	129
*A. sudafricanum* (unresolved)	28	0.01998	67
*D. capensis*	8	0.08592	101
*D. natalensis*	6	0.01881	21

**Figure 5.**
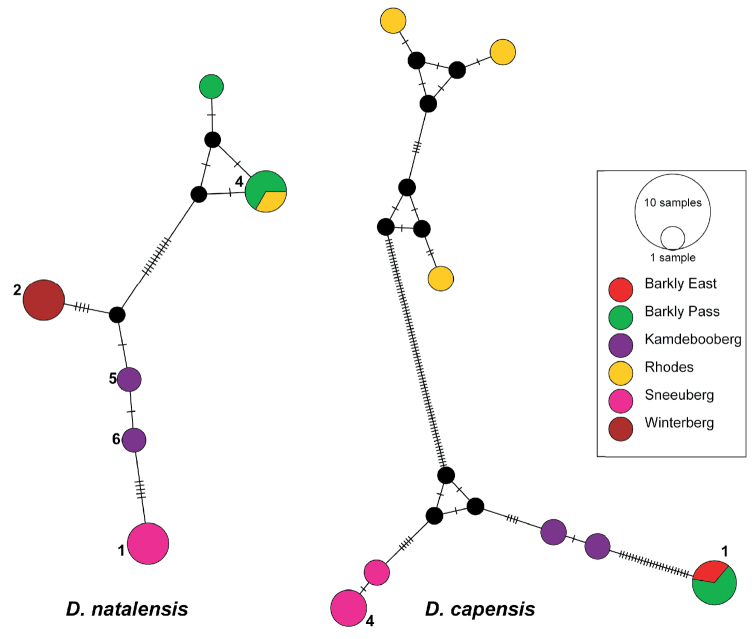
Median-joining networks of *D. natalensis* and *D. capensis* based on COI haplotypes generated in this study. The network was estimated using the median-joining algorithm in PoPArt v.1.7 with epsilon = 0. Each circle represents a different haplotype and the size of a circle correlates with number of individuals belonging to that given haplotype. Only haplotypes found in more than one sample are numbered. Colours indicate the geographic origin of sequences; black dots indicate unsampled or extinct haplotypes.

The MJN analyses for *D. capensis* retrieved eight haplotypes (Hd = 0.9273, S = 101), six of which were singletons and *D. natalensis* retrieved six haplotypes (Hd = 0.8636, S = 21) including three singletons (Fig. 5). Haplotypes were largely site-restricted for both species with the exception of Haplotype 1 (*N* = 3) for *D. capensis* and Haplotype 4 (*N* = 3) for *D. natalensis* (study areas Barkly Pass and Rhodes), which are both found in the Eastern Cape Drakensberg main mountain block in the Escarpment (Fig. 4). Both networks show many missing mutational steps between haplotypes grouped by locality, which could result from undersampling or haplotype filtering.

Nucleotide diversities (P_i_) are reported in Table 3 and are not interpreted further because the small sample sizes for *Demoreptus* spp. make the estimates imprecise. Neutrality tests (Tajima’s D and Fu’s F_S_) were not significant for *A. sudafricanum*, *D. capensis* or *D. natalensis* indicating that the nucleotide patterns of variation are consistent with the neutral theory of evolution. Fu’s F_S_ statistic for the widespread grade of *A. sudafricanum* was negative (F_S_ = –11.544) and significant (P < 0.02), indicating a recent population expansion (Table 3). The Fu’s F_S_ statistics for *D. capensis* and *D. natalensis* were positive, indicating a deficiency of alleles as expected from a population bottleneck, but they were not significant and need a larger sample size to confirm these results.

The AMOVA results for *A. sudafricanum* revealed that over all localities, 52.33% of the total variance was explained by variation among populations (df = 10, Va = 12.073) while 47.67% (df = 75, Vb = 10.998) was explained by variation within populations (Table 4). A similar result was found with the widespread grade of *A. sudafricanum*, with 39.43% of the total variance explained by variation among populations (df = 5, Va = 2.238) and 60.57% (df = 28, Va = 3.438) explained by variation within populations. In contrast, the AMOVAs for the habitat-restricted species, *D. capensis* and *D. natalensis*, indicated a higher proportion of variation among populations: 94.83% (df = 4, Va = 24.950) and 95.39% (df = 4, Va = 5.423), respectively (Table 4). The total variance explained by variation within populations was only 5.17% (df = 6, Vb = 1.361) for *D. capensis* and 4.61% (df = 7, Vb = 0.262) for *D. natalensis*.

The measure of population differentiation due to genetic structure (F_ST_) was much lower for *A. sudafricanum* compared to the *Demoreptus* species (Table 4). The widespread grade for *A. sudafricanum* had a very low F_ST_ value of 0.39 while *D. natalensis* and *D. capensis* had very high F_ST_ values of over 0.94 (Table 4).

**Table 4.** One-level AMOVA results for *A. sudafricanum*, *D. capensis* and *D. natalensis* showing percentage variation among and within populations and the fixation index (F_ST_). Significant p-values (< 0.05) are set in bold.

Species/clade	% variation		F_ST_
	Among	Within	
*A. sudafricanum*	52.33	47.67	**0.52327**
*A. sudafricanum* unresolved group	39.43	60.57	**0.39426**
*D. capensis*	94.83	5.17	**0.94827**
*D. natalensis*	95.39	4.61	**0.95393**

## Discussion

This study considered evidence of the phylogenetic structure of three species of Baetidae corresponding to two different habitat requirements. Results indicate that habitat-restricted *Demoreptus* species have greater maternal genetic structure than widespread *A. sudafricanum*, showing notable genetic differentiation associated with geographic localities and catchments. This is evident from the haplotype networks in a MJN analysis, F_ST_ values from an AMOVA and the phylogeographical structure indicated by phylogenetic trees.

Phylogeographical structure of habitat generalist, *A. sudafricanum* retrieved six distinct, well-supported clades and one widespread grade of individuals from widespread (Escarpment and non-Escarpment) sites across the sampling area. *Afroptilum sudafricanum* was best represented with a haplotype network (Fig. 3), particularly for the widespread grade as the samples have evolved over such a short time that ancestral and descendant haplotypes exist concurrently, and so it remains unresolved in the hierarchical tree. The species occupies a range of habitats from still to flowing rivers. Remarkably, shared haplotypes (Haps 10, 17, and 19) were identified between Escarpment and non-Escarpment sites, some over 300 km apart (Hap 10), across various mountain chains and differing in altitude by over 900 m (Fig. 4). The genetic differentiation within *A. sudafricanum* is not attributed to purely geographic location or catchments. Most clades seen in both the hierarchical trees and haplotype networks include sites that are widely spread across sampled catchments and mountain blocks, with the exception of one clade that occurs only in the Eastern Cape Drakensberg (Rhodes and Barkly East). Even if *A. sudafricanum* is treated as a species complex and assessed for mitochondrial genetic differentiation, results indicate low divergence between populations, suggesting that gene flow is not particularly limited within catchments and across the geographic range. Although mayflies are traditionally thought to have limited dispersal ability due to weak flight and short adult lifespans (Brittain and Sartori 2003, Monaghan et al. 2005, Gattolliat et al. 2008), some mitochondrial clades within *A. sudafricanum* are remarkably widespread. These results support studies indicating that long-distance dispersal is in fact more prevalent in mayflies than previously thought (Monaghan et al. 2005, Gattolliat and Staniczek 2011, Pereira-da-Conceicoa et al. 2012, Vuataz et al. 2013, Rutschmann et al. 2016).

The habitat specialist species, *D. natalensis* and *D. capensis* are rheophilic and found on rock faces associated with waterfalls and large bedrock sections in shallow but fast-flowing sections of mountain streams. Analyses indicate restricted gene flow over distance and across catchments, a possible consequence of isolation by habitat limitations in mountainous areas. Distinct clades retrieved from phylogenetic analyses show a close association with geographic locality. *Demoreptus natalensis* returned clades and haplotypes exclusive to Sneeuberg and Winterberg areas; the Eastern Cape Drakensberg clade included two study areas (Barkly Pass and Rhodes areas); and Kamdebooberg was unresolved. *Demoreptus capensis* had a similar result, but the Rhodes area returned a separated clade with a well-supported, long branch. Suggestively, the samples of *A. sudafricanum* and *D. capensis* collected at Rhodes both occupy long branches in their respective phylogenetic analyses (Figs 2, 3). These sites are from the highest regions of the study (2600 m.a.s.l.) on the slopes of Ben MacDhui. This may indicate a historical isolation event or an accelerated local rate of molecular evolution (perhaps through faster fixation in smaller populations) responsible for the pattern observed.

Preliminary re-examinations indicate morphological differences between *D. capensis* from Rhodes and *D. capensis* from other localities, and between *D. natalensis* from Barkly East and *D. natalensis* from other localities (HMBJ, pers. obs.); these characters will be documented in a subsequent taxonomic study. Other areas in the Drakensberg range in KwaZulu-Natal and Lesotho should be sampled to investigate the range of this mitochondrial clade and whether it occurs throughout high altitude, mountainous areas. A caveat is that the *Demoreptus* population analyses involve limited sample sizes from few localities, which can produce misleading clustering (Phiri and Daniels 2014, Hinojosa et al. 2019), and that sampling more localities can address this concern (Phiri and Daniels 2014). Furthermore, mitochondrial genes are inherited asexually and maternally, and may represent gene flow differently from sexually-inherited, recombining nuclear genes (Hinojosa et al. 2019), so quantifying nuclear gene diversity is also necessary to clarify this situation.

Previous studies on South African species have found genetic differentiation according to catchments in both animals with limited dispersal ability (Wishart and Hughes 2001, 2003, Daniels et al. 2009, McDonald and Daniels 2012, Tolley et al. 2014, Barber-James and Pereira-da-Conceicoa 2016) and terrestrial insects with high vagility (Price et al. 2007, 2010). The unexpected limited dispersal potential of cicadas was attributed to their habitat philopatry (Price et al. 2010) and host-plant specificity (Price et al. 2007). Similarly, *D. natalensis* and *D. capensis* are restricted by their habitat, and subsequently show high levels of genetic differentiation. Similar limitations to gene flow have been found in various other mountain-restricted aquatic insects (Hughes et al. 2003, Wishart and Hughes 2003, Finn et al. 2006, Lehrian et al. 2010).

The high support values for some geographically localised clades within *A. sudafricanum* and the two *Demoreptus* species could indicate the presence of cryptic species or local haplotype filtering and mutation due to protracted isolation (Hinojosa et al. 2019). Mountain-dwelling populations are often fragmented and under-sampled (Phiri and Daniels 2013), and the reported low diversity of Baetidae in most areas of Africa has been attributed to the lack of data and comprehensive analysis of material collected by taxonomists (Gattolliat et al. 2008). Intensive sampling over large geographical ranges usually results in the discovery of numerous new taxa and the extension of distribution ranges (Gattolliat et al. 2008). Cryptic taxa are not uncommon in aquatic insects (Wishart and Hughes 2003, 200, Pereira-da-Conceicoa et al. 2012). Since the 1980s there has been an exponential increase in the number of studies on cryptic species, partly due to the introduction of the PCR, which resulted in the increasing availability of DNA sequences (Bickford et al. 2007). Molecular (DNA) methods are valuable in resolving morphologically cryptic lineages and have been used extensively in discriminating species with few or no morphological differences (Jackson and Resh 1998, Rutschmann et al. 2014, Leys et al. 2016, Tenchini et al. 2018). Within the Ephemeroptera, cryptic lineages have been discovered in numerous families through electrophoretic studies (Sweeney and Funk 1991, Zloty et al. 1993, Funk and Sweeney 1994) and, more recently, DNA sequence data (Williams et al. 2006, Ståhls and Savolainen 2008, Pereira-da-Conceicoa et al. 2012).

The observed deep haplotype divergences in all three species studied and the recent population expansion in *A. sudafricanum* may be explained by possible Quaternary glaciation in the Drakensberg area, where small glaciers formed as low as 2100 m on south-facing slopes (Lewis and Hanvey 1993, Lewis and Illgner 2001, Grab 2002, Mills and Grab 2005, Lewis 2011, Mills et al. 2012). Small remnant populations in non-glaciated areas at high altitude would have been isolated for some time which may explain the long branch patterns seen in *D. capensis* and *A. sudafricanum* for high altitude populations from Rhodes in the Eastern Cape Drakensberg. Glaciation would exacerbate the difficulty of finding suitable habitats more for *Demoreptus* spp. than for *A. sudafricanum*, which can find suitable habitats at lower altitudes). However, the evidence available for this niche glaciation is considered by some as ambiguous and unclear (Osmaston and Harrison 2005). Cyclical climate changes from the Pleistocene to present interglacial (Dingle and Rogers 1972, Siesser and Dingle 1981) could have resulted in historic population fluctuations including expansions, bottlenecks, drift and allele fixation (especially for *A. sudafricanum*).

However, because they are asexually and maternally inherited, strongly divergent haplotypes that originated in relict populations may not reflect contemporary mating pattern if those isolated populations’ ranges subsequently expand to restore potential panmixis (Hinojosa et al. 2019). More samples and an investigation of nuclear genetic diversity are necessary to get any further resolution into the patterns observed.

### Perspectives

These results help to illuminate some of the evolutionary processes occurring in mayfly species and highlight the effect of habitat-specificity on haplotype diversity and partitioning within a species. While all three species have qualitatively similar levels of dispersal potential in terms of flight, they show differences in gene flow, suggesting that other processes, such as species-specific habitat requirements, may contribute to genetic population structure. These results have implications for the conservation of riverine organisms, the reintroduction of locally extinct taxa and the rehabilitation of disturbed environments (Jump et al. 2009, Razgour et al. 2019).

In South Africa, it is legislated that catchments are used as management units (Republic of South Africa 1998). Previous studies on the genetic population structure of winged aquatic insects in South Africa have further supported the use of catchments as units for conservation (Wishart and Hughes 2003, Wishart et al. 2003, Price et al. 2010). The results found here for *D. capensis* and *D. natalensis* further highlight the genetic distinctiveness of populations between catchments, further corroborating the value of using catchments in conservation, management and legislative frameworks. These genetically distinct populations form an important component in the evolutionary legacy of a species. Therefore, the development of inter-basin water transfer schemes poses a threat to both *D. capensis* and *D. natalensis* and many other species by potentially connecting historically isolated and genetically distinct populations (Snaddon and Davies 1998, Davies et al. 2000)

In addition, dispersal among adjacent catchments has implications for the recovery of lotic systems following disturbance (Wishart and Davies 2003, Bellingan et al. 2019, Razgour et al. 2019). These factors should be considered in the development of strategies for the conservation of aquatic biodiversity (Wishart 2000, Thieme et al. 2007, Castello et al. 2013, Bellingan et al. 2019), and most particularly for high altitude catchments.

This study highlights the importance for future studies on community structure, biodiversity, and biomonitoring, where the taxonomic accuracy of species identification is crucial (Hajibabaei et al. 2016). The identification of possible cryptic species in *A. sudafricanum* and new species of *Demoreptus* affect the field of aquatic research in South Africa. Mayflies form a very important component of applied aquatic biology, particularly biomonitoring, the presence of cryptic taxa is being discovered at an increasing rate and poses challenges for some aquatic ecosystem monitoring methods. With bioassessment methods gaining increasing popularity, a detailed understanding of commonly collected species will aid in further development of assessment methods and clarify species identification (Delić et al. 2017, Suh et al. 2019). In addition, a deeper understanding of evolutionary processes and gene flow with regard to commonly occurring mayfly taxa contributes to broader research on ecosystem functioning and environmental processes. The utility of DNA barcoding for elucidating such phenomena is already proven (Jackson and Resh 1998, Plaisance et al. 2009, Raupach and Radulovici 2015) and widely used, with new technologies allowing for the rapid assessment of biodiversity using DNA metabarcoding (Pavan-Kumar et al. 2015, Elbrecht et al. 2017, Daravath et al. 2018, Alvarez-Yela et al. 2019). This approach to rapid biodiversity assessment has the potential to revolutionise and streamline management and conservation practices by providing detailed data for informed decision- and policy-making.

## Supplementary materials

Supplementary material 1 List of GenBank sequence accession numbers for each sample Link: https://doi.org/10.3897/zookeys.936.38587.suppl1 Data type molecular data https://binary.pensoft.net/file/414730This dataset is made available under the Open Database License (http://opendatacommons.org/licenses/odbl/1.0/). The Open Database License (ODbL) is a license agreement intended to allow users to freely share, modify, and use this Dataset while maintaining this same freedom for others, provided that the original source and author(s) are credited.Chantal L. Taylor, Nigel P. Barker, Helen M. Barber-James, Martin H. Villet, Lyndall L. Pereira-da-Conceicoa
